# Supplementary material for: A slowly cleaved viral signal peptide acts as a protein-integral immune evasion domain
Source: Nat Commun. 2021 Apr 6;12:2061. doi: 10.1038/s41467-021-21983-x (PMC8024260; doi:10.1038/s41467-021-21983-x)
Supplement: Supplementary file 1 — Supplementary Information [file 41467_2021_21983_MOESM1_ESM.pdf]

Supplementary Information

a

```
  1   milwspstcsffwhwcliavsvlssrsKESLRLSWSSDESSASSSSRICPLSNSKSVRLP   60
 61   QYPRGFGDVSGYRVSSSVSECYVQHGVLVAAWLVRGNFSDTAPRAYGTWGNERSATHFKV  120
121   GAPQLENDGALRYETELPQVDARLSYVMLTVYPCSACNRSVLHCRPASRLPWLPLRVTPS  180
181   DLERLFAERRYLTFLYVVLVQFVKHVALFSFGVQVACCVYLRWIRPWVRGRHRATGRTSR  240
241   EEEAKDD                                                       247
```

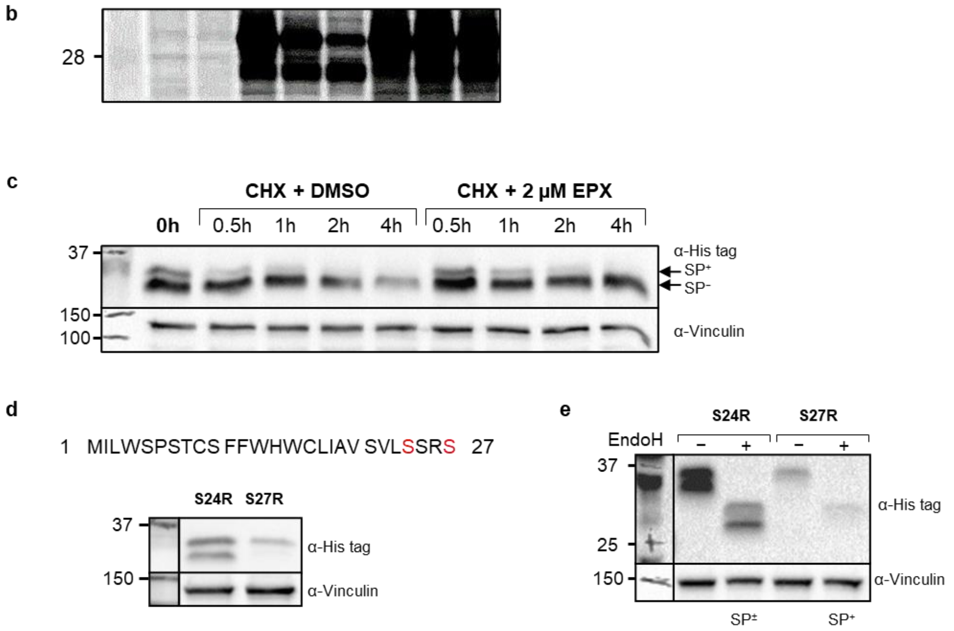

Figure S1, associated with figure 1. US9 contains a slowly-cleaved SP. (a) US9 amino acid sequence (accession P09729 from HCMV strain AD169) with the following domains annotated: SP (lowercase characters); serine-rich compositionally biased area (red font); Ig-like domain (underlined); transmembrane domain (bold). Transmembrane domain location was predicted by the Phobius algorithm (http://phobius.sbc.su.se/), other domains are based on Uniprot annotation (https://www.uniprot.org/). (b) Ladder visualized in a higher contrast image of the pulse-chase shown in Fig. 1b. (c) RKO MICA*008-HA cells expressing US9 were treated with cycloheximide (CHX), combined with a DMSO control or with epoxomicin (EPX). At the indicated time points, lysates were prepared and blotted using anti-His tag for US9 visualization and anti-vinculin as loading control. US9 forms with or without the SP ($SP^{+/-}$) are indicated. A single experiment was performed. (d) The US9 SP sequence with two

potential serine signal peptide cleavage sites highlighted in red. Two serine-to-arginine mutants were constructed (S24R and S27R) and expressed in RKO MICA*008-HA. Transfectants were lysed and immunoblotted. Anti-His tag was used to visualize protein expression, with anti-vinculin as loading control. Different segments of the same gel are shown together for clarity. Representative of three independent experiments. (d) Lysates prepared as in (c) were untreated (−) or digested with endoH (+) and then blotted. Anti-His tag was used to visualize protein expression, with anti-vinculin as loading control. Annotation beneath the panel indicates US9 forms with, or both with and without the SP ($SP^{+/\pm}$). Different segments of the same gel are shown together for clarity. A single experiment was performed.

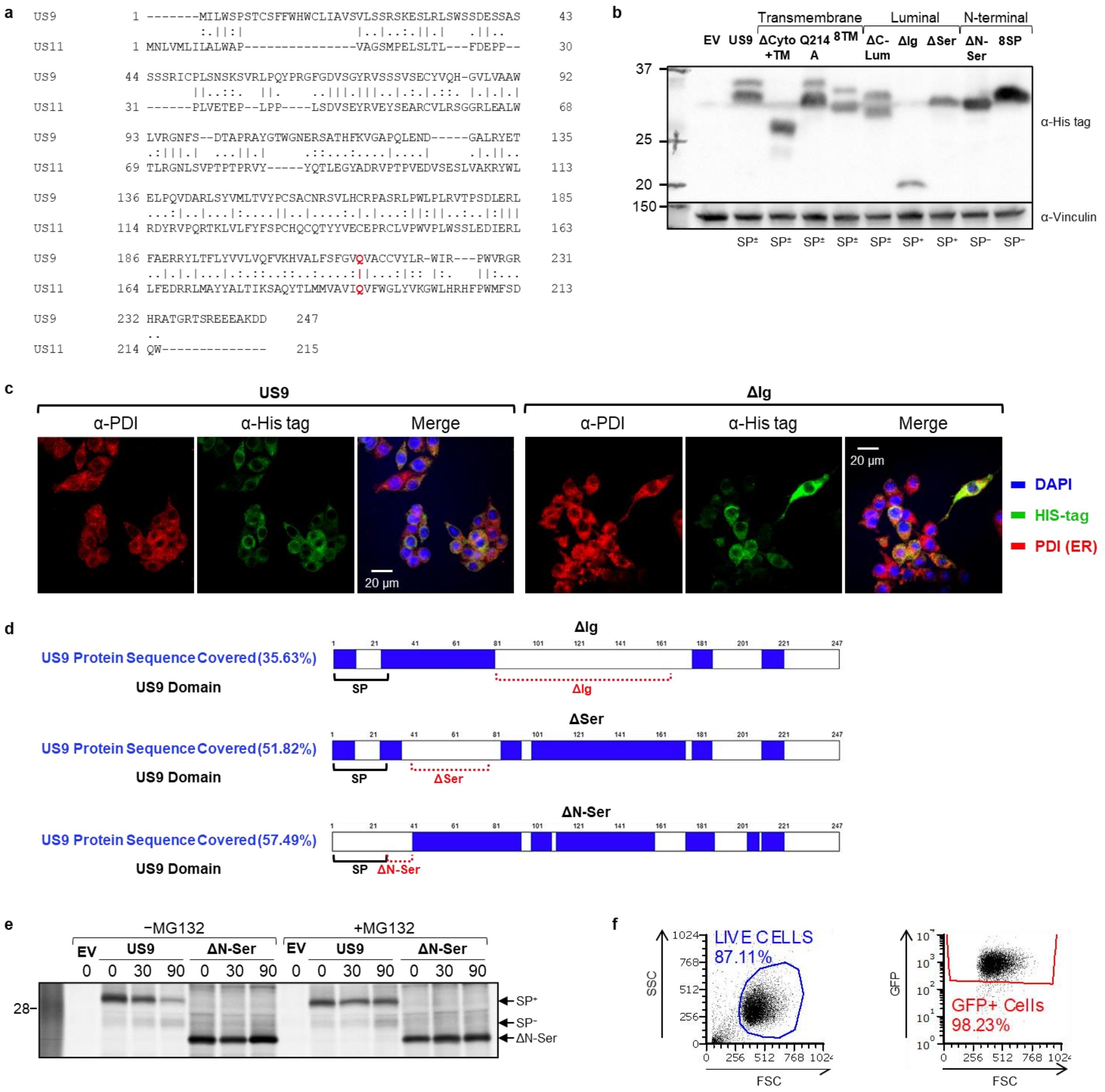

Figure S2, associated with figures 2-3. Design, expression, localization and SP status of the US9 mutants. (a) Sequence alignment of US9 (accession P09729 from strain AD169) and US11 (accession P09727 from strain AD169). Highlighted in red is the conserved transmembrane glutamine residue (Q214 in US9 and Q192 in US11) which in US11 is essential for Derlin1 recruitment[1–3]. (b) US9 mutants described in Fig. 2 were expressed in RKO MICA*008-HA cells. Cells were lysed and immunoblotted using anti-His tag to visualize protein expression, with anti-vinculin as loading control. Mutants are grouped by mutated region (indicated). Annotation beneath the panel indicates US9 forms with, without,

or both with and without the SP ($SP^{+/-/\pm}$). Different panels depict separate gels. Representative of three independent repeats. (c) Intracellular localization of US9 and of the ΔIg mutant demonstrated by confocal microscopy in RKO MICA*008-HA cells, stained with an anti-His tag antibody (green) and an anti-protein disulfide isomerase (PDI) antibody (ER marker; red). Nuclei were stained with DAPI (blue). Representative of two independent experiments. (d) Graphic representation of US9 protein sequence coverage detected by mass spectrometry analysis of the indicated US9 deletion mutants (deleted sequences shown by dashed red lines). Coverage % of WT US9 sequence and US9 domains are indicated. See also Supplementary Data 3-5. (e) RKO MICA*008-HA cells expressing EV, US9 or ΔN-Ser were labeled for 20 minutes (pulse) and then chased in the absence (−) or presence (+) of 10 μM MG132. Digitonin-lysates were prepared at the indicated chase times (in minutes). An anti-His tag IP was performed. All samples were digested with endoH. Representative of three independent experiments. (f) Gating strategy for the flow cytometry staining in Fig. 3a. First, RKO MICA*008-HA EV live cells were gated based on forward scatter (FSC) and side scatter (SSC) to exclude cellular debris, and then GFP^+ cells were gated within a daughter gate. A similar strategy was used for all other stainings in this work.

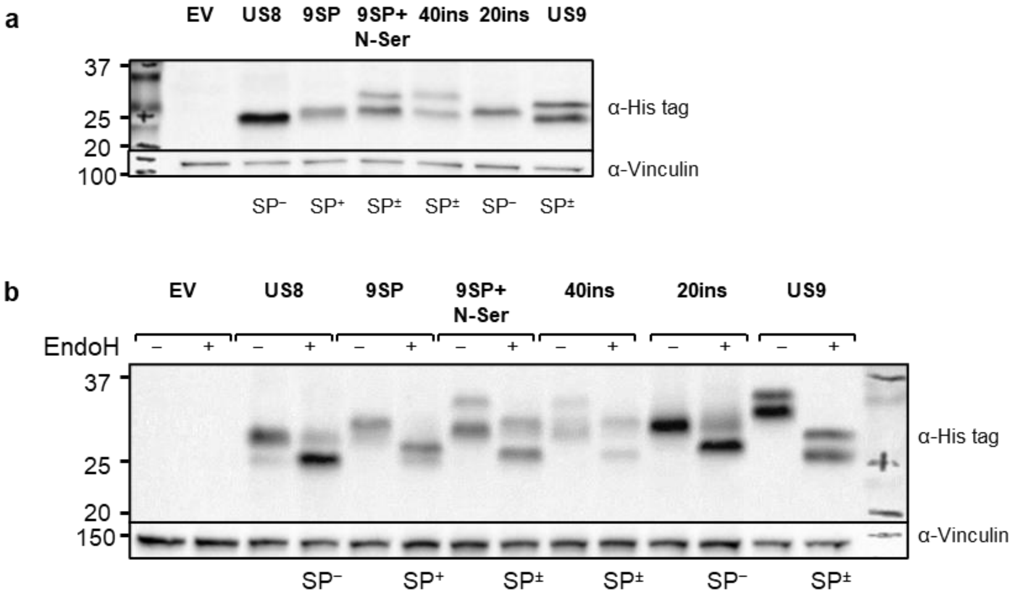

Figure S3, associated with figure 4. SP Status and localization of the US8 mutants. (a) The indicated US8 mutants described in Fig. 4 were expressed in RKO MICA*008-HA cells, and lysates were prepared, digested with PNGaseF and then immunoblotted. Anti-His tag was used to visualize expression, with anti-vinculin as loading control. Annotation beneath the panel indicates US8 constructs with, without, or both with and without the US9 SP ($SP^{+/-/\pm}$). Representative of three independent experiments. (b) Lysates prepared from the indicated transfectants were untreated (−) or digested with endoH (+) and then blotted using anti-His tag, with anti-vinculin as loading control. Annotation beneath the panel indicates US8 constructs with, without, or both with and without the US9 SP ($SP^{+/-/\pm}$). Representative of three independent experiments.

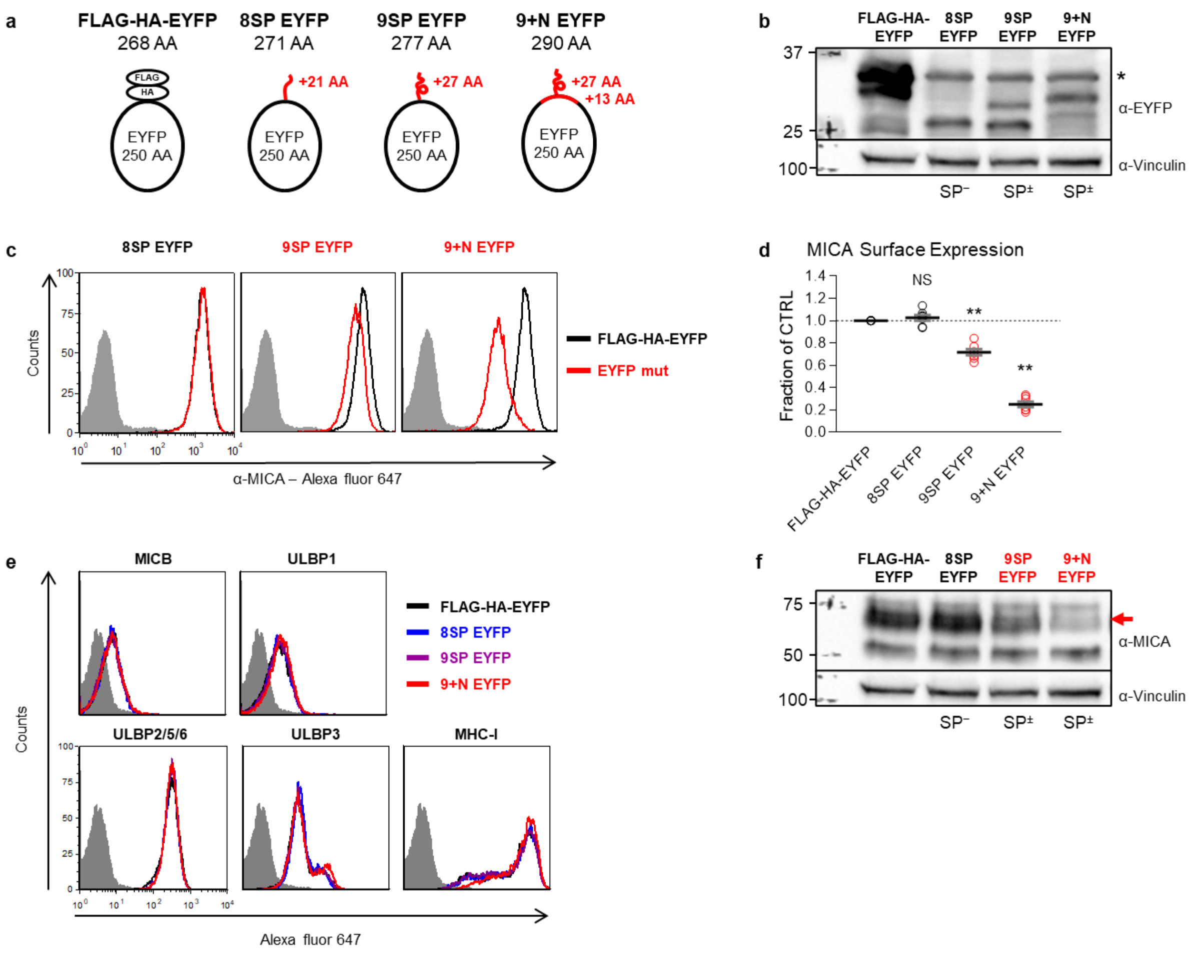

Figure S4, associated with figure 4. The US9 SP is sufficient for targeting MICA*008 to degradation when attached to EYFP. (a) Schematic representation of EYFP chimeric constructs stably transfected into RKO MICA*008 cells. N-terminal tags were removed from FLAG-HA-tagged EYFP protein (named FLAG-HA-EYFP), and replaced with the US8 SP, the US9 SP or the US9 SP and N-Ser domain (respectively named 8SP, 9SP and 9+N). The length of EYFP, of the inserted domains and total construct length are indicated. (b) Cells expressing the indicated transfectants were lysed and immunoblotted with anti-EYFP to assess protein expression, with anti-vinculin as loading control. Annotation beneath the panel indicates EYFP constructs without, or both with and without the US9 SP ($SP^{-/\pm}$). * - nonspecific band. Representative of two independent experiments. (c) MICA*008 surface expression in the indicated transfectants. Red font highlights gain-of-function. Gray-filled histograms represent secondary antibody staining of FLAG-HA-EYFP. (d) Quantification of

MICA surface expression shown in (c). MFIs were normalized to the MFI of FLAG-HA-EYFP transfectants. Data show mean ±SEM for seven independent experiments. A one-way ANOVA was performed with a significant effect at the $p<0.05$ level for all conditions [$F_{(3,24)} = 258.651$, $p = 2.11 \cdot 10^{-18}$]. A post-hoc Dunnett's test was used to compare the MFI for FLAG-HA-EYFP (indicated by the dashed line) to that of each mutant. ** = $P<0.01$. NS – non-significant. Red circles highlight gain-of-function. (e) Surface expression of the indicated proteins was measured by flow cytometry in the indicated transfectants. Gray-filled histograms represent secondary antibody staining of the FLAG-HA-EYFP control. Shown is one of four independent experiments. (f) Lysates obtained from the indicated cells were blotted using anti-MICA as part of the same experiment shown in (b) and sharing the same anti-vinculin loading control. Red font highlights gain-of-function, and the red arrow indicates post-ER MICA*008. Annotation beneath the panel indicates EYFP constructs without, or both with and without the US9 SP ($SP^{-/\pm}$). One of two independent experiments is shown. See also the Raw Data file for experimental data and full statistics.

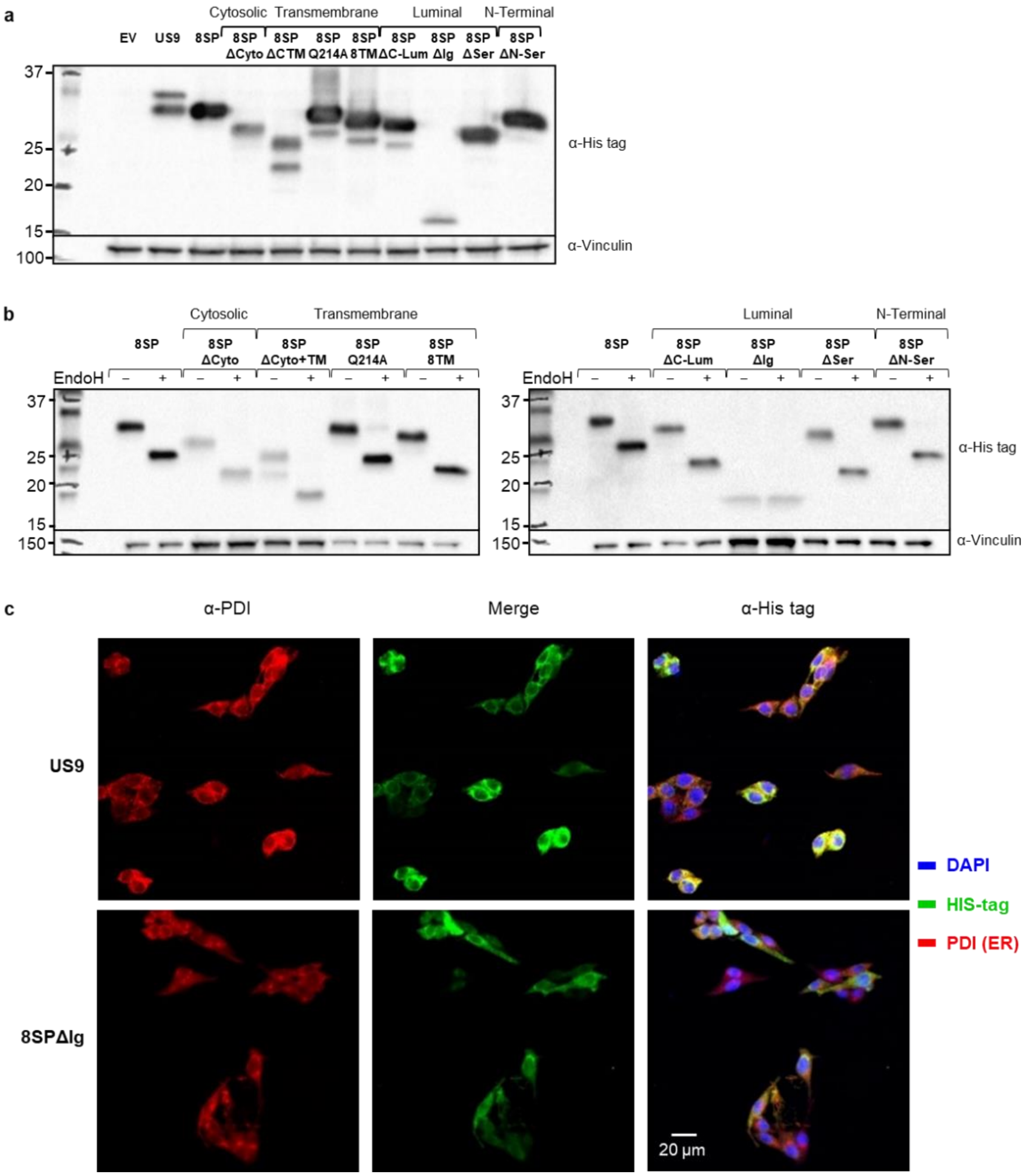

Figure S5, associated with figure 5. Expression levels and ER localization of the US9 double mutants. (a) US9 double mutants described in Fig. 5 were expressed in RKO MICA*008-HA cells, and lysates were prepared and then blotted. Anti-His tag was used to visualize expression, with anti-vinculin as loading control. Mutants are grouped by the mutated region (indicated). Representative of four independent experiments. (b) Lysates prepared from cells expressing the indicated US9 double mutants were untreated (−) or

digested with endoH (+) and then blotted using anti-His tag and anti-vinculin as loading control. Mutants are grouped by mutated region (indicated). Panels depict different gels. Representative of four independent experiments, except for 8SPΔCyto and 8SPΔN-Ser for which a single experiment was performed. (c) Intracellular localization of US9 and of the 8SPΔIg mutant demonstrated by confocal microscopy in RKO MICA*008 cells, stained with an anti-His tag antibody (green) and an anti-protein disulfide isomerase (PDI) antibody (ER marker; red). Nuclei were stained with DAPI (blue). A single experiment was performed.

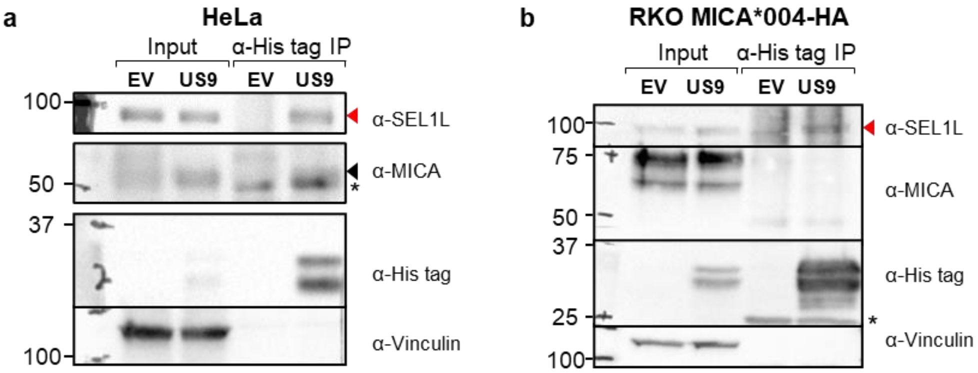

Figure S6, associated with figure 6. US9 binds endogenous MICA*008 and binds SEL1L even in MICA*008's absence. (a) HeLa cells, which endogenously express MICA*008, were transduced with an EV or with US9 and lysed. An immunoprecipitation (IP) was performed using anti-HIS tag antibody. Eluates were divided in three and run separately (shown by different panels) to facilitate blotting. Anti-SEL1L (top panel), and-MICA (middle panel) and anti-His tag (bottom panel) were used to visualize protein co-precipitation. Anti-vinculin (bottom panel) served as input loading control. Arrows indicate bands which specifically co-precipitated with US9 (red – SEL1L; black – MICA*008). * - antibody heavy chain. Representative of two independent experiments. (b) Lysates prepared from RKO MICA*004-HA cells expressing EV or US9 were immunoprecipitated using anti-His tag antibody. An immunoblot was performed using anti-His tag, anti-MICA and anti-SEL1L to visualize protein co-precipitation, with anti-vinculin as input loading control. Red arrow indicates SEL1L co-precipitation with US9. * - antibody light chain. Representative of three independent experiments.

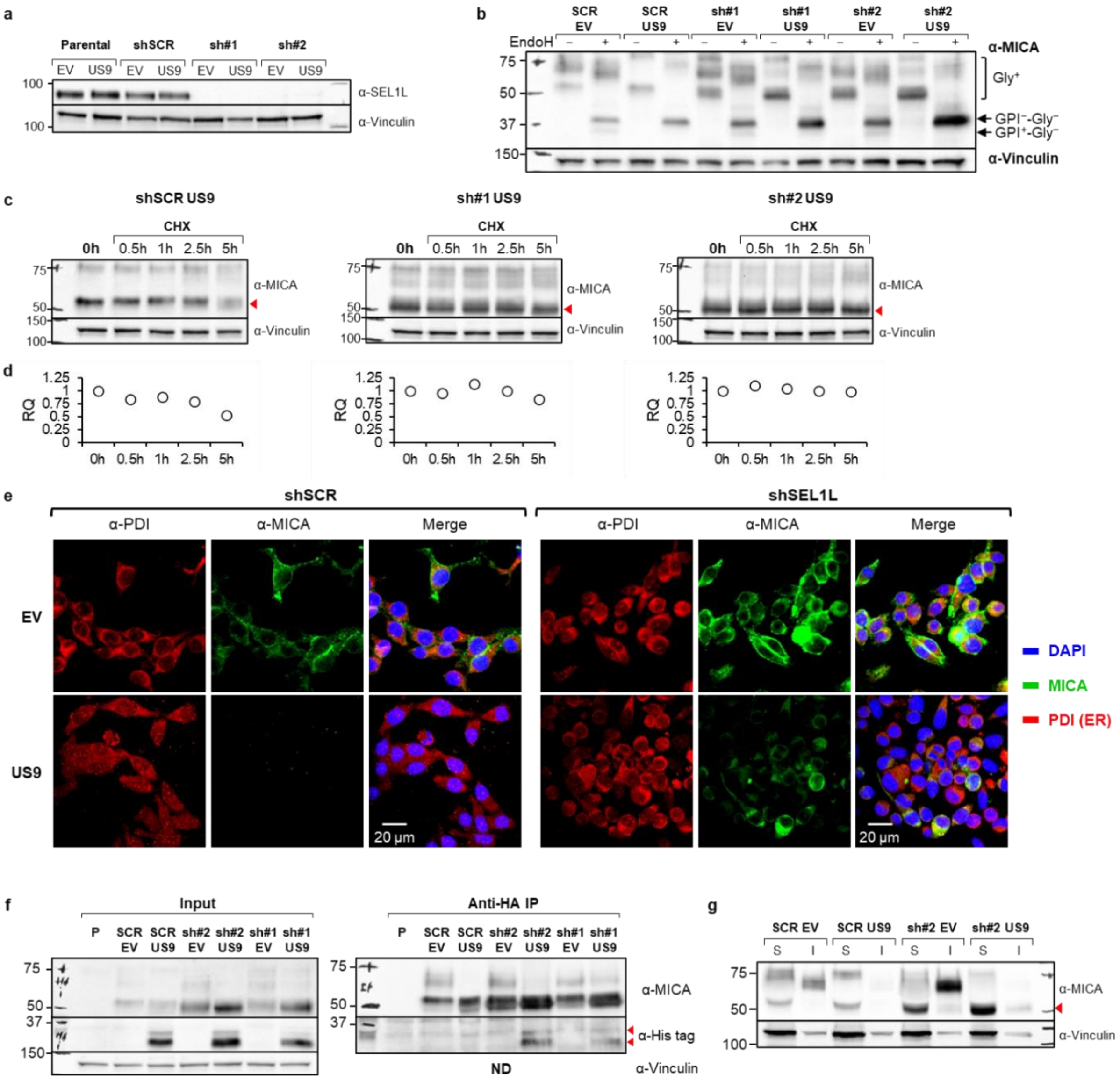

Figure S7, associated with figures 7-8. US9 induces MICA*008 maturation arrest in SEL1L-depleted cells. (a) RKO MICA*008-HA cells expressing shScrambled, shSEL1L#1 or shSEL1L#2 (shSCR, sh#1 and sh#2, respectively) were co-transduced with an EV or with US9. Lysates were blotted using anti-SEL1L for validation of knockdown efficiency, with anti-vinculin as loading control. Representative of three independent experiments. (b) Lysates from the indicated cells were then left untreated (−) or digested with endoH (+). Blotting was performed using anti-MICA, with anti-vinculin as loading control. Glycosylated MICA*008 forms are annotated (Gly^{+}), and arrows indicate deglycosylated MICA*008 forms with or

without the GPI anchor (GPI$^{+/-}$-Gly^{-}). A single experiment was performed. (c-d) RKO MICA*008-HA US9 cells transduced with the indicated shRNA constructs were treated with cycloheximide (CHX). At the indicated time points (h - hours), lysates were prepared, and blotted (c) using anti-MICA and anti-vinculin (loading control). Red arrows show the ER-resident form of MICA. (d) Quantification of the ER-resident form of MICA*008 shown in (c), relative to the 0h control and normalized to the loading control. RQ – relative quantity. Representative of three independent experiments for shSCR and sh#2, a single experiment was performed for sh#1. (e) Confocal microscopy of the indicated cells, stained with an anti-MICA antibody (green) and an anti-protein disulfide isomerase (PDI) antibody (ER marker; red). Nuclei were stained with DAPI (blue). A single experiment was performed. (f) An immunoprecipitation (IP) was performed on lysates prepared from the indicated cells using anti-HA tag antibody. Eluates were blotted using anti-His tag, anti-MICA and anti-SEL1L to visualize protein co-precipitation, with anti-vinculin as input loading control. Left panel – input; right panel – IP. Red arrows indicate US9 bands which specifically co-precipitated with MICA. * - antibody light chain. ND – not detected. Representative of two independent experiments. (g) RKO MICA*008-HA cells expressing the indicated shRNAs and co-transduced with an EV or with US9 were fractionated into detergent soluble (S) and insoluble (I) fractions, which were then immunoblotted using anti-MICA and anti-vinculin as loading control. Red arrow shows the ER-resident form of MICA*008. A single experiment was performed. See also the Raw Data file for experimental data.

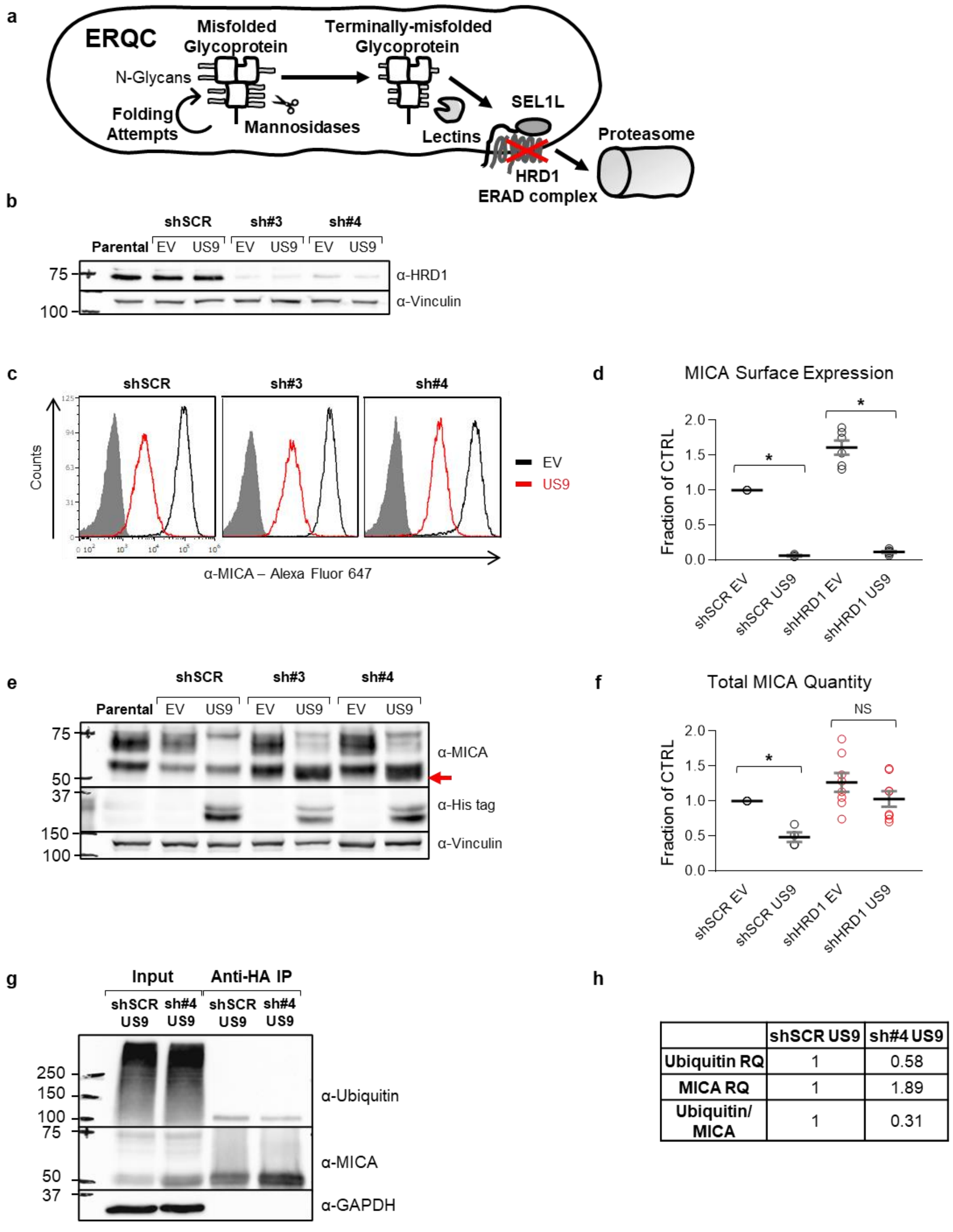

	shSCR US9	sh#4 US9
Ubiquitin RQ	1	0.58
MICA RQ	1	1.89
Ubiquitin/ MICA	1	0.31

Figure S8, associated with figures 7-8. HRD1 is required for US9-mediated MICA*008 degradation. (a) Schematic representation of the effect of HRD1 knockdown (red X) on the ER quality control (ERQC) pathway. HRD1 knockdown should cause the accumulation of terminally glycosylation-trimmed misfolded glycoproteins. (b) Lysates from RKO

MICA*008-HA cells transduced with shScrambled, shHRD1#3 and shHRD1#4 (shSCR, sh#3 and sh#4, respectively) co-expressing an EV or US9, were blotted using anti-HRD1 for validation of knockdown efficiency, with anti-vinculin as loading control. Representative of three independent experiments. (c) RKO MICA*008-HA cells expressing the indicated shRNAs and co-transduced with an EV or with US9 were stained for MICA surface expression. Gray-filled histograms represent secondary antibody staining of the EV control. (d) Quantification of MICA surface expression shown in (c). MFIs were normalized to the shSCR EV MFI. Data show mean ±SEM for three independent experiments. Two-tailed unequal variance Student's T-tests were used to compare EV vs. US9 surface MICA levels in shSCR cells or in pooled shHRD1 cells (shSCR $p = 1.25 \cdot 10^{-4}$, shHRD1 $p = 2.26 \cdot 10^{-5}$). Sidak's correction was used to determine a corrected alpha level, * = $P<0.0252$. (e) Lysates obtained from the indicated cells were blotted using anti-MICA and anti-vinculin as loading control. The red arrow indicates glycosylation-trimmed MICA*008. (f) Total MICA*008 protein levels shown in (e) were quantified relative to the shSCR EV control and normalized to the loading control. Data show mean ±SEM for four independent experiments. Two-tailed unequal variance Student's T-tests were used to compare EV vs. US9 surface MICA levels in shSCR cells or in pooled shHRD1 cells (shSCR $p = 4.89 \cdot 10^{-3}$, shSEL1L $p = 0.19$). Sidak's correction was used to determine a corrected alpha level, * = $P<0.0252$. NS = not significant. (g-h) RKO MICA*008-HA cells expressing shSCR or sh#4 were co-transduced with US9 and treated with the proteasomal inhibitor EPX. (g) Lysates were prepared and immunoprecipitated using anti-HA tag. Immunoblotting was performed using anti-MICA and anti-ubiquitin, with anti-GAPDH as input loading control. The red arrow indicates polyubiquitinylated MICA*008. (h) Relative quantification (RQ) of immunoprecipitated MICA*008 forms shown in (g). Representative of two independent experiments. See also the Raw Data file for experimental data.

Supplementary Table 1. Primers and templates used for cloning

Construct name	Step	Forward primer	Reverse primer	Template
ΔCyto+TM	1/1	ccgcggccgcgccgccaccatgatcctgtggtccccgtc	gggatcctcaatgatgatgatgatgatgtgtcaggtagcgtctctccg	US9-HIS
Q214A	1/3	ccgcggccgcgccgccaccatgatcctgtggtccccgtc	gcaggctaccaggacaccgaagct	US9-HIS
	2/3	agcttcggtgtcctggtagcctgc	gggatcctcaatgatgatgatgatgatgatcgtctttagcctcttcttccc	US9-HIS
	3/3	ccgcggccgcgccgccaccatgatcctgtggtccccgtc	gggatcctcaatgatgatgatgatgatgatcgtctttagcctcttcttccc	Reactions #1+#2
8TM	1/6	ccgcggccgcgccgccaccatgatcctgtggtccccgtc	cgaccccgagtgtcaggtagcgtctctccg	US9-HIS
	2/6	gctacctgacactcggggtcgtgatcgccat	cagggacgtatggccagcacgtatcccaaca	US8-HIS
	3/6	cgtgctggccatacgtccctgggtccgaggt	gggatcctcaatgatgatgatgatgatgatcgtctttagcctcttcttccc	US9-HIS
	4/6	ccgcggccgcgccgccaccatgatcctgtggtccccgtc	cagggacgtatggccagcacgtatcccaaca	Reactions #1+#2
	5/6	gctacctgacactcggggtcgtgatcgccat	gggatcctcaatgatgatgatgatgatgatcgtctttagcctcttcttccc	Reactions #2+#3
	6/6	ccgcggccgcgccgccaccatgatcctgtggtccccgtc	gggatcctcaatgatgatgatgatgatgatcgtctttagcctcttcttccc	Reactions #4+#5
ΔC-lum	1/3	ccgcggccgcgccgccaccatgatcctgtggtccccgtc	catagaggaaggacgccggccggcaatgg	US9-HIS
	2/3	ccggcgtccttcctctatgtggtactggtgc	gggatcctcaatgatgatgatgatgatgatcgtctttagcctcttcttccc	US9-HIS
	3/3	ccgcggccgcgccgccaccatgatcctgtggtccccgtc	gggatcctcaatgatgatgatgatgatgatcgtctttagcctcttcttccc	Reactions #1+#2
ΔIg	1/3	ccgcggccgcgccgccaccatgatcctgtggtccccgtc	agggcagcctggacacggaactggagacac	US9-HIS
	2/3	gttccgtgtccaggctgccctggttgccgc	gggatcctcaatgatgatgatgatgatgatcgtctttagcctcttcttccc	US9-HIS
	3/3	ccgcggccgcgccgccaccatgatcctgtggtccccgtc	gggatcctcaatgatgatgatgatgatgatcgtctttagcctcttcttccc	Reactions #1+#2
ΔSer	1/3	ccgcggccgcgccgccaccatgatcctgtggtccccgtc	cgtaacattccgactcgtcgctggaccac	US9-HIS
	2/3	gcgacgagtcg*gaatgttacgtccagcacggg*	gggatcctcaatgatgatgatgatgatgatcgtctttagcctcttcttccc	US9-HIS
	3/3	ccgcggccgcgccgccaccatgatcctgtggtccccgtc	gggatcctcaatgatgatgatgatgatgatcgtctttagcctcttcttccc	Reactions #1+#2
ΔN-Ser	1/3	ccgcggccgcgccgccaccatgatcctgtggtccccgtc	aggacgcagaggagcggctcgagagtacact	US9-HIS
	2/3	gagccgctcctctgcgtcctcgtcgtctcgt	gggatcctcaatgatgatgatgatgatgatcgtctttagcctcttcttccc	US9-HIS
	3/3	ccgcggccgcgccgccaccatgatcctgtggtccccgtc	gggatcctcaatgatgatgatgatgatgatcgtctttagcctcttcttccc	Reactions #1+#2
8SP	1/3	ccgcggccgcgccgccaccatgaggcggtggctgcgc	gcgactccttggcgtgagccagcgttaccc	US8-HIS
	2/3	ggctcacgccaaggagtcgctccggttgtcg	gggatcctcaatgatgatgatgatgatgatcgtctttagcctcttcttccc	US9-HIS
	3/3	ccgcggccgcgccgccaccatgaggcggtggctgcgc	gggatcctcaatgatgatgatgatgatgatcgtctttagcctcttcttccc	Reactions #1+#2
8SPΔCyto	1/1	ccgcggccgcgccgccaccatgaggcggtggctgcgc	gggatcctcaatgatgatgatgatgatgccacctcaggtacacacagc	8SP
8SPΔCyto+TM	1/1	ccgcggccgcgccgccaccatgaggcggtggctgcgc	gggatcctcaatgatgatgatgatgatgtgtcaggtagcgtctctccg	8SP
8SPQ214A	1/3	ccgcggccgcgccgccaccatgaggcggtggctgcgc	gcgactccttggcgtgagccagcgttaccc	US8-HIS
	2/3	ggctcacgccaaggagtcgctccggttgtcg	gggatcctcaatgatgatgatgatgatgatcgtctttagcctcttcttccc	Q214A
	3/3	ccgcggccgcgccgccaccatgaggcggtggctgcgc	gggatcctcaatgatgatgatgatgatgatcgtctttagcctcttcttccc	Reactions #1+#2

8SP8TM	1/3	ccgcggccgcgccgccaccatgaggcggtggctgcgc	gcgactccttggcgtgagccagcgttaccc	US8-HIS
	2/3	ggctcacgccaaggagtcgctccggttgtcg	gggatcctcaatgatgatgatgatgatgatcgtctttagcctcttcttccc	8TM
	3/3	ccgcggccgcgccgccaccatgaggcggtggctgcgc	gggatcctcaatgatgatgatgatgatgatcgtctttagcctcttcttccc	Reactions #1+#2
8SPΔC-Lum	1/3	ccgcggccgcgccgccaccatgaggcggtggctgcgc	gcgactccttggcgtgagccagcgttaccc	US8-HIS
	2/3	ggctcacgccaaggagtcgctccggttgtcg	gggatcctcaatgatgatgatgatgatgatcgtctttagcctcttcttccc	ΔC-lum
	3/3	ccgcggccgcgccgccaccatgaggcggtggctgcgc	gggatcctcaatgatgatgatgatgatgatcgtctttagcctcttcttccc	Reactions #1+#2
8SPΔIg	1/3	ccgcggccgcgccgccaccatgaggcggtggctgcgc	gcgactccttggcgtgagccagcgttaccc	US8-HIS
	2/3	ggctcacgccaaggagtcgctccggttgtcg	gggatcctcaatgatgatgatgatgatgatcgtctttagcctcttcttccc	ΔIg
	3/3	ccgcggccgcgccgccaccatgaggcggtggctgcgc	gggatcctcaatgatgatgatgatgatgatcgtctttagcctcttcttccc	Reactions #1+#2
8SPΔSer	1/3	ccgcggccgcgccgccaccatgaggcggtggctgcgc	gcgactccttggcgtgagccagcgttaccc	US8-HIS
	2/3	ggctcacgccaaggagtcgctccggttgtcg	gggatcctcaatgatgatgatgatgatgatcgtctttagcctcttcttccc	ΔSer
	3/3	ccgcggccgcgccgccaccatgaggcggtggctgcgc	gggatcctcaatgatgatgatgatgatgatcgtctttagcctcttcttccc	Reactions #1+#2
8SPΔN-Ser	1/3	ccgcggccgcgccgccaccatgaggcggtggctgcgc	aggacgcagaggcgtgagccagcgttaccc	US8-HIS
	2/3	ggctcacgcctctgcgtcctcgtcgtctcgt	gggatcctcaatgatgatgatgatgatgatcgtctttagcctcttcttccc	US9-HIS
	3/3	ccgcggccgcgccgccaccatgaggcggtggctgcgc	gggatcctcaatgatgatgatgatgatgatcgtctttagcctcttcttccc	Reactions #1+#2
S24R	1/3	ccgcggccgcgccgccaccatgatcctgtggtccccgtc	tggagcggctgcggagtacacttactgcgatcagacac	US9-HIS
	2/3	aagtgtactccgcagccgctccaaggagtcg	gggatcctcaatgatgatgatgatgatgatcgtctttagcctcttcttccc	US9-HIS
	3/3	ccgcggccgcgccgccaccatgatcctgtggtccccgtc	gggatcctcaatgatgatgatgatgatgatcgtctttagcctcttcttccc	Reactions #1+#2
S27R	1/3	ccgcggccgcgccgccaccatgatcctgtggtccccgtc	gcgactccttgcggcggctcgagagtacacttac	US9-HIS
	2/3	ctcgagccgccgcaaggagtcgctccggttgt	gggatcctcaatgatgatgatgatgatgatcgtctttagcctcttcttccc	US9-HIS
	3/3	ccgcggccgcgccgccaccatgatcctgtggtccccgtc	gggatcctcaatgatgatgatgatgatgatcgtctttagcctcttcttccc	Reactions #1+#2
9SP	1/3	ccgcggccgcgccgccaccatgatcctgtggtccccgtc	aggggttgccggagcggctcgagagtacact	US9-HIS
	2/3	gagccgctccggcaacccctatgaggacgac	gggatcctcaatgatgatgatgatgatgacataaggacttttcacacttttgg	US8-HIS
	3/3	ccgcggccgcgccgccaccatgatcctgtggtccccgtc	gggatcctcaatgatgatgatgatgatgacataaggacttttcacacttttgg	Reactions #1+#2
9SP+NSer	1/3	ccgcggccgcgccgccaccatgatcctgtggtccccgtc	aggggttgcccgactcgtcgctggaccac	US9-HIS
	2/3	cgacgagtcgggcaacccctatgaggacga	gggatcctcaatgatgatgatgatgatgacataaggacttttcacacttttgg	US8-HIS
	3/3	ccgcggccgcgccgccaccatgatcctgtggtccccgtc	gggatcctcaatgatgatgatgatgatgacataaggacttttcacacttttgg	Reactions #1+#2
20ins	1/6	ccgcggccgcgccgccaccatgaggcggtggctgcgc	agagtacactggcgtgagccagcgttaccc	US8-HIS
	2/6	ggctcacgccagtgtactctcgagccgctcca	aggggttgcccgactcgtcgctggaccac	US9-HIS
	3/6	cgacgagtcgggcaacccctatgaggacga	gggatcctcaatgatgatgatgatgatgacataaggacttttcacacttttgg	US8-HIS
	4/6	ccgcggccgcgccgccaccatgaggcggtggctgcgc	aggggttgcccgactcgtcgctggaccac	Reactions #1+#2
	5/6	ggctcacgccagtgtactctcgagccgctcca	gggatcctcaatgatgatgatgatgatgacataaggacttttcacacttttgg	Reactions #2+#3
	6/6	ccgcggccgcgccgccaccatgaggcggtggctgcgc	gggatcctcaatgatgatgatgatgatgacataaggacttttcacacttttgg	Reactions

				#4+#5
40ins	1/3	ccgcggccgcgccgccaccatgaggcggtggctgcgc	acaggatcatggcgtgagccagcgttaccc	US8-HIS
	2/3	ggctcacgccatgatcctgtggtccccgtc	gggatcctcaatgatgatgatgatgatgacataaggacttttcacacttttgg	9SP+NSer
	3/3	cgacgagtcgggcaacccctatgaggacga	gggatcctcaatgatgatgatgatgatgacataaggacttttcacacttttgg	Reactions #1+#2
8SP EYFP	1/1	gatatcgccggccgccatgaggcggtggctgcgc	agcggccgctggcgtgagccagcgttaccc	US8-HIS
9SP EYFP	1/1	cgatatcgccggccgccatgatcctgtggtccccgtc	agcggccgctggagcggctcgagagtacact	US9-HIS
9SP+N-Ser EYFP	1/1	cgatatcgccggccgccatgatcctgtggtccccgtc	agcggccgctcgactcgtcgctggaccac	US9-HIS

Supplementary References

1. Lilley, B. N. & Ploegh, H. L. A membrane protein required for dislocation of misfolded proteins from the ER. *Nature* **429**, 834–840 (2004).

2. Ye, Y., Shibata, Y., Yun, C., Ron, D. & Rapoport, T. A. A membrane protein complex mediates retro-translocation from the ER lumen into the cytosol. *Nature* **429**, 841–847 (2004).

3. Lilley, B. N., Tortorella, D. & Ploegh, H. L. Dislocation of a type I membrane protein requires interactions between membrane-spanning segments within the lipid bilayer. *Mol. Biol. Cell* **14**, 3690–3698 (2003).
