# Supplementary material for: A slowly cleaved viral signal peptide acts as a protein-integral immune evasion domain
Source: Nat Commun. 2021 Apr 6;12:2061. doi: 10.1038/s41467-021-21983-x (PMC8024260; doi:10.1038/s41467-021-21983-x)
Supplement: Supplementary file 3 — Description of Additional Supplementary Files [file 41467_2021_21983_MOESM3_ESM.pdf]

Description of Additional Supplementary Files

File Name: Supplementary Data 1
Description: Mass spectrometry data of SP+ US9, associated with Fig. 1.

File Name: Supplementary Data 2
Description: Mass spectrometry data of SP− US9, associated with Fig. 1.

File Name: Supplementary Data 3
Description: Mass spectrometry data of ΔIg US9, associated with Fig. S2.

File Name: Supplementary Data 4
Description: Mass spectrometry data of ΔSer US9, associated with Fig. S2.

File Name: Supplementary Data 5
Description: Mass spectrometry data of ΔN-Ser US9, associated with Fig. S2.

File Name: Supplementary Data 6
Description: Mass spectrometry data of SEL1L, associated with Fig. 6.
