# Supplementary material for: A slowly cleaved viral signal peptide acts as a protein-integral immune evasion domain
Source: Nat Commun. 2021 Apr 6;12:2061. doi: 10.1038/s41467-021-21983-x (PMC8024260; doi:10.1038/s41467-021-21983-x)
Supplement: Supplementary file 10 — Reporting Summary [file 41467_2021_21983_MOESM10_ESM.pdf]

nature research

Corresponding author(s): Prof. Ofer Mandelboim

Last updated by author(s): 15/02/2021

Reporting Summary

Nature Research wishes to improve the reproducibility of the work that we publish. This form provides structure for consistency and transparency in reporting. For further information on Nature Research policies, see our Editorial Policies and the Editorial Policy Checklist.

Statistics

For all statistical analyses, confirm that the following items are present in the figure legend, table legend, main text, or Methods section.

n/a	Confirmed	
☐	☒	The exact sample size (n) for each experimental group/condition, given as a discrete number and unit of measurement
☐	☒	A statement on whether measurements were taken from distinct samples or whether the same sample was measured repeatedly
☐	☒	The statistical test(s) used AND whether they are one- or two-sided<br>*Only common tests should be described solely by name; describe more complex techniques in the Methods section.*
☐	☒	A description of all covariates tested
☐	☒	A description of any assumptions or corrections, such as tests of normality and adjustment for multiple comparisons
☐	☒	A full description of the statistical parameters including central tendency (e.g. means) or other basic estimates (e.g. regression coefficient) AND variation (e.g. standard deviation) or associated estimates of uncertainty (e.g. confidence intervals)
☐	☒	For null hypothesis testing, the test statistic (e.g. F, t, r) with confidence intervals, effect sizes, degrees of freedom and P value noted<br>*Give P values as exact values whenever suitable.*
☒	☐	For Bayesian analysis, information on the choice of priors and Markov chain Monte Carlo settings
☒	☐	For hierarchical and complex designs, identification of the appropriate level for tests and full reporting of outcomes
☐	☒	Estimates of effect sizes (e.g. Cohen's d, Pearson's r), indicating how they were calculated

Our web collection on statistics for biologists contains articles on many of the points above.

Software and code

Policy information about availability of computer code

Data collection	For Olympus FluoView FV1000 software (immunofluorescence), FCS Express version 6 software (flow cytometry), ImageLab software (immunoblot), ImageJ (autoradiography), MaxQuant software and Discoverer software (mass spectrometry and proteomics)
Data analysis	For statistical analyses: Real Statistics Resource Pack software (Release 5.4), GraphPad Prism version 8

For manuscripts utilizing custom algorithms or software that are central to the research but not yet described in published literature, software must be made available to editors and reviewers. We strongly encourage code deposition in a community repository (e.g. GitHub). See the Nature Research guidelines for submitting code & software for further information.

Data

Policy information about availability of data

All manuscripts must include a data availability statement. This statement should provide the following information, where applicable:

- Accession codes, unique identifiers, or web links for publicly available datasets
- A list of figures that have associated raw data
- A description of any restrictions on data availability

The data underlying Figs. 1C, 3B, 3D, 4D, 5C, 7C, 7E, 8A, 9C, 9E, and Supplementary Figs. 4D, 7D, 8D and 8F, including full statistical analyses, are provided as a Raw Data file. Remaining data is available in the article, Supplementary Data files or available from the authors upon request

April 2020

Field-specific reporting

Please select the one below that is the best fit for your research. If you are not sure, read the appropriate sections before making your selection.

- [x] Life sciences
- [ ] Behavioural & social sciences
- [ ] Ecological, evolutionary & environmental sciences

For a reference copy of the document with all sections, see nature.com/documents/nr-reporting-summary-flat.pdf

Life sciences study design

All studies must disclose on these points even when the disclosure is negative.

Sample size	no sample size calculation was performed
Data exclusions	no data were excluded
Replication	Biological replicates were performed with fresh cells from at least two separate transfections/transductions.
Randomization	Not relevant - all experiments were performed in vitro.
Blinding	Not relevant - all experiments were performed in vitro.

Reporting for specific materials, systems and methods

We require information from authors about some types of materials, experimental systems and methods used in many studies. Here, indicate whether each material, system or method listed is relevant to your study. If you are not sure if a list item applies to your research, read the appropriate section before selecting a response.

Materials & experimental systems

n/a	Involved in the study	
[ ]	[x]	Antibodies
[ ]	[x]	Eukaryotic cell lines
[x]	[ ]	Palaeontology and archaeology
[x]	[ ]	Animals and other organisms
[x]	[ ]	Human research participants
[x]	[ ]	Clinical data
[x]	[ ]	Dual use research of concern

Methods

n/a	Involved in the study	
[x]	[ ]	ChIP-seq
[ ]	[x]	Flow cytometry
[x]	[ ]	MRI-based neuroimaging

Antibodies

Antibodies used

Anti-MICB (clone 236511, R&D Systems), anti-ULBP1 (clone 170818, R&D Systems), anti-ULBP2/5/6 (clone 165903, R&D Systems), anti-ULBP3 (clone 166514, R&D Systems), and anti-MHC class I (W6/32 hybridoma) primary antibodies were used for flow cytometry. The anti-PDI (rabbit polyclonal, Abcam, Cambridge, MA), anti-MICA (clone 159227, R&D Systems) and anti-HIS tag (clone AD1.1.10, R&D systems) primary antibodies were used for immunofluorescence.

The following primary antibodies were used for western immunoblotting: anti-HIS tag (clone AD1.1.10, R&D systems), anti-MICA (clone EPR6568, Abcam), anti-SEL1L (rabbit polyclonal to N-terminus, Sigma-Aldrich), EYFP cross reactive anti-GFP (rabbit polyclonal, ab290, Abcam), anti-HRD1 (rabbit polyclonal, NB100-2526, Novus Biologicals), anti-ubiquitin (clone FK2, Merck-Millipore), anti-GAPDH (clone 6C5, Santa Cruz) and anti-vinculin (clone EPR8185, Abcam).

Validation

Antibodies were used for manufacturer-validated applications, the hybridoma W6/32 was extensively characterized and used in multiple publications.

Eukaryotic cell lines

Policy information about cell lines

Cell line source(s)	ATCC
Authentication	Cells were obtained from the ATCC and were not authenticated again following receipt.
Mycoplasma contamination	Cell lines were routinely tested for mycoplasma contamination and were negative.

April 2020

Commonly misidentified lines (See ICLAC register)	N/A

Flow Cytometry

Plots

Confirm that:

- [x] The axis labels state the marker and fluorochrome used (e.g. CD4-FITC).
- [x] The axis scales are clearly visible. Include numbers along axes only for bottom left plot of group (a 'group' is an analysis of identical markers).
- [x] All plots are contour plots with outliers or pseudocolor plots.
- [x] A numerical value for number of cells or percentage (with statistics) is provided.

Methodology

Sample preparation	Cells were plated at equal densities and incubated overnight. Resuspended cells were incubated on ice for 1 hour with the primary antibody at a concentration of 0.2 µg per well. The cells were then incubated for 30 minutes on ice with the appropriate secondary antibody at a concentration of 0.75 µg per well. In all experiments using cells transduced with a GFP-expressing lentivirus or transfected with EYFP, the histograms are gated on the live GFP/EYFP+ population. 10,000 live cells were acquired from each sample.
Instrument	FACSCalibur flow cytometer (BD Biosciences) and CytoFLEX flow cytometer (Beckman Coulter)
Software	FACS Express version 6
Cell population abundance	Only cell populations with >90% GFP+ fractions were used for experiments
Gating strategy	Live cells were gated based on forward scatter (FSC) and side scatter (SSC) to exclude cellular debris, and then GFP+ cells were gated within a daughter gate (Fig. S2F).

- [x] Tick this box to confirm that a figure exemplifying the gating strategy is provided in the Supplementary Information.
